# Supplementary figures and images for: Human Salivary Microbiota Diversity According to Ethnicity, Sex, TRPV1 Variants and Sensitivity to Capsaicin
Source: Int J Mol Sci. 2024 Oct 29;25(21):11585. doi: 10.3390/ijms252111585 (PMC11546822; doi:10.3390/ijms252111585)

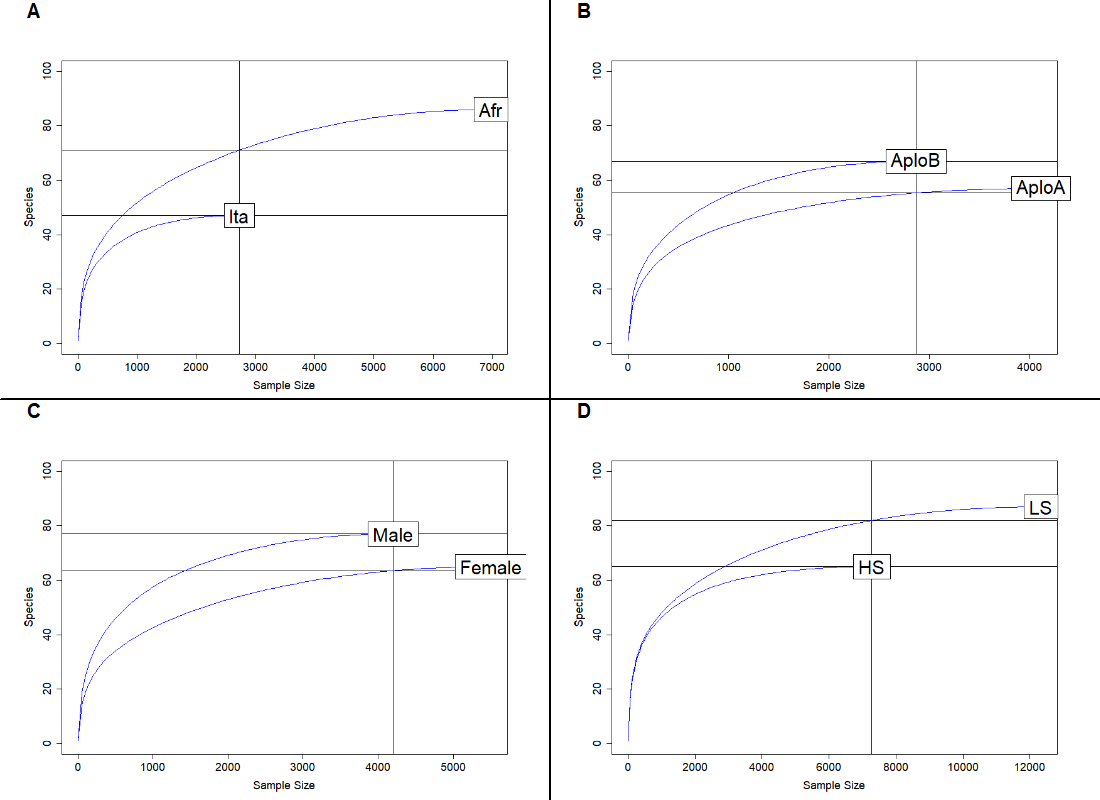

Supplement: Supplementary file 1 [file ijms-25-11585-s001.zip › Figure S1.png]
